# Reducing wastewater nitrogen loading by >90% with carbon-amended septic systems: A field demonstration in Barnstable (Cape Cod), Massachusetts

**DOI:** 10.1016/j.jenvman.2024.122737

**Published:** 2024-10-11

**Authors:** Laura E. Erban, Sara K. Wigginton, Brian Baumgaertel, Bryan Horsley, Timothy D. McCobb, Zenas Crocker, Scott Horsley, Timothy R. Gleason

**Affiliations:** a U.S. Environmental Protection Agency, Office of Research and Development, Narragansett, RI, USA; b Massachusetts Alternative Septic System Test Center, Sandwich, MA, USA; c U.S. Geological Survey, New England Water Science Center, Northborough, MA, USA; d Barnstable Clean Water Coalition, Osterville, MA, USA; e Horsley Consulting, Cotuit, MA, USA

**Keywords:** Wastewater, Groundwater, Nitrogen, OWTS, I/A septic systems, Woodchip bioreactor

## Abstract

Onsite wastewater treatment systems (OWTS) are a major source of excess nutrients and co-pollutants in watersheds across the United States. In Barnstable County (Cape Cod), Massachusetts, effluent from septic systems and cesspools contributes approximately 80% of the controllable reactive nitrogen (N) load to numerous impaired estuaries and degrades water quality in the region’s sole source aquifer, streams and ponds. In unsewered areas, wastewater N loads could be reduced substantially by Innovative/Alternative (I/A) septic systems designed for enhanced removal. Use, however, has been partly limited by the availability of high performing, cost effective options, while conventional septic systems continue to be installed in watersheds with well documented N impairments. This paper describes the strategic replacement of residential OWTS with two I/A models that incorporate woodchip bioreactors to enhance N removal. Systems were installed at 14 neighboring homes in Barnstable, MA, and monitored for field performance. Influent and effluent were sampled monthly and analyzed for N and phosphorus (P), among other water quality indicators. Flow to each system was continuously metered to estimate nutrient loads. Results from the first 25 months of monitoring for 13 systems with at least a full year of data are presented in terms of 1) reductions in nutrient concentrations and mass loads and 2) reliability of the systems for meeting a performance goal of total N (TN) < 10 mg/L. Discussion supports consideration of where and how these technologies may be successfully used to manage excess N in sensitive watersheds.

## 1. Introduction

Onsite wastewater treatment systems (OWTS) pose risks to water quality and human and environmental health that have been acknowledged for decades. A 1977 Report to Congress identified septic systems and cesspools as responsible for regional groundwater contamination, and areas with more than 40 systems per square mile (or 1 per 16 acres) as being at risk (USEPA, 1977). Initially intended for low density rural settings, OWTS, commonly known as septic systems, have proliferated since the second world war in higher density subdivisions and closely spaced lots in suburban and urban areas (Scalf et al., 1977; USEPA, 1977). The proximity of functioning or hydraulically failing OWTS to wells and surface water bodies can lead to contamination with human fecal indicators and pathogens and cause outbreaks of pathogenic disease (Yates, 1985; Beller et al., 1997; Carroll et al., 2006; Wallender et al., 2014; Murphy et al., 2020).

Septic system effluent also contains excess nutrients and co-pollutants linked to other direct and indirect hazards. Regions with coarse sediments or fractured rock are particularly vulnerable since water infiltrates rapidly (Bouma et al., 1972; Canter and Knox, 1984; LeBlanc, 1984; USDA, 1994; DeSimone and Howes, 1998; Karathanasis et al., 2006; Rivett et al., 2008; Gill et al., 2009). Prime examples are found in coastal watersheds of the northeast United States (US), where dense populations with limited sewer access live on glacially deposited sand and gravel aquifers with high permeability and low capacity for contaminant attenuation. Groundwater is often the sole source of drinking water, and it transports pollutants to streams, ponds, and estuaries. Tourism, recreation, and fisheries are drivers of “blue”, or marine-based, economies and ways of life that are adversely impacted when coastal ecosystems are overloaded with undertreated effluent.

Effluent from conventional septic systems is enriched in nitrogen (N). N is highly mobile in groundwater as nitrate (NO_3_^−^) which is federally regulated in drinking water at a level of 10 mg/L NO_3_-N, set to reduce the risk of blue baby syndrome in infants, Lower concentrations may increase risks of colorectal cancer and thyroid disease, among other conditions, in the general population (Ward et al., 2018) and can also damage aquatic ecosystems. N limits primary production in marine waters, and, along with phosphorus (P) in freshwater systems, contributes to algal blooms (Bricker et al., 1999; Howarth et al., 2002; Conley et al., 2009; Paerl et al., 2016). Blooms of cyanobacteria, micro- and macroalgae reduce water clarity, leading to loss of benthic vegetation and habitat over time. Nuisance algal blooms in water bodies are unappealing to recreational users. Harmful algal blooms (HABs) cause low dissolved oxygen events and fish kills during decomposition. Some HABs produce toxins that can interfere with drinking water supply or prompt closures of areas used for swimming and shellfishing.

Estuaries across Cape Cod (i.e., Barnstable County, Massachusetts) are impaired by excess N, and decentralized wastewater disposal is the primary source. More than half of these water bodies, over 30 in total, have Total Maximum Daily Loads (TMDLs). TMDLs, required by the Clean Water Act’s (CWA) Section 303(d) for impaired waters, provide N pollution reduction targets for restoration. Cape-wide, estuarine TMDLs call for ~50% reduction in N loading from onsite wastewater sources (CCC, 2015). Under CWA Section 208, stakeholders have engaged in extensive deliberation and planning towards attainment of water quality goals (Perry et al., 2020). In some areas with higher density and load reduction targets, sewers and centralized treatment capacity are being expanded. Many locations will not be able to connect to a sewer for decades, if ever. Enhanced onsite wastewater treatment, among other innovations, can reduce or prevent pollutant loading in unsewered areas with or without a TMDL.

Septic systems designed for N removal enhance the microbially mediated, coupled processes of nitrification and denitrification. A conventional septic system includes a tank for solids and a leach field to disperse and infiltrate liquid effluent. Aerobic conditions in the unsaturated leach field allow autotrophic nitrifiers to convert ammonium (NH_4_^+^) to nitrate (NO_3_^−^). Concurrently, alkalinity and organic carbon in the wastewater are consumed. Denitrification completes N removal by converting NO_3_^−^ in water to N_2_ gas. It is largely driven by facultative heterotrophic bacteria given access to sufficient organic carbon for nitrate reduction and deprived of oxygen, conditions that are typically limiting in a leach field or underlying aquifer. To enhance denitrification prior to discharge, nitrified effluent can be recirculated to the septic tank, itself anoxic and carbon-rich, or routed to an alternate anoxic reactor with supplemental carbon. Traditional biological N removal uses the former approach, but three process configurations that apply the anoxic and aerobic reactors in either order or in combination, with discrete internal zones, are commonly used in different wastewater treatment contexts (Oakley et al., 2010).

Bioreactors made of woodchips have long been used as a low-cost technique for mitigating nitrate in agricultural ditches and in the sub-surface, as permeable reactive barriers, including to treat septic effluent plumes (Robertson et al., 2000; Robertson et al., 2008; Schipper et al., 2010; Addy et al., 2016). Incorporating lignocellulosic materials like woodchips or sawdust in a septic system simply relocates the reactive media to the source for more effective treatment, reducing N by 80–90% or more in experimental and field trials (Robertson et al., 2000; Robertson et al., 2008; Rich, 2008; Oakley et al., 2010; FLDOH, 2015;Heufelder, 2019; Gobler et al., 2021; Wigginton et al., 2021). Woodchips are well suited to biofilm growth and preservation of hydraulic conductivity and serve as a slow-release carbon source that is both sufficiently labile and durable for long-term (15 years or more) N removal, with low rates of material consumption when continuously saturated (Robertson et al., 2000, 2008; Robertson, 2010; Lopez-Ponnada et al., 2017; Lepine et al., 2021). Septic systems with woodchip bioreactors have also mitigated some organic contaminants in pharmaceuticals and personal care products (Gobler et al., 2021), which are among a large set co-pollutants found widely in the Cape’s ground, surface waters and tap waters (Rudel, 1998; Standley, 2008; Schaider et al., 2014; Bradley et al., 2021).

Septic systems designed for enhanced N removal vary in complexity, performance, and availability in different states. In Massachusetts, they are referred to as Innovative/Alternative (I/A). Manufacturers have historically targeted a performance goal of 19 mg/L total nitrogen (TN) in effluent that is not low enough for the TMDL-specified reduction targets in some watersheds. Recent amendments, in 2023, to the state environmental code (310 CMR 15, or “Title 5”) set a new goal of 10 mg/L TN for best available nitrogen-reducing technologies (BANRT). Field testing is necessary to thoroughly evaluate performance under representative operating conditions. Massachusetts requires 50 installations and 3 years of monitoring before an I/A septic system can be considered for general use approval. Without high performance and general use approval, local jurisdictions can be reluctant to require them. Adoption by homeowners further depends on affordability, installation and maintenance procedures, aesthetics, perceived risks, and personal values (Rudman et al., 2023). At present, few technologies that can meet the more stringent 10 mg/L TN performance goal are readily available and acceptable to users.

In this demonstration study, partners with the U.S. Environmental Protection Agency’s Office of Research and Development (EPA ORD), the U.S. Geological Survey (USGS), the Massachusetts Alternative Septic System Test Center (MASSTC), and the Barnstable Clean Water Coalition (BCWC), worked with The Nature Conservancy (TNC) and with local government (Town of Barnstable), businesses and residents to replace existing OWTS with best available N-reducing technologies in early stages of permitting. Over a 25-month period, the team evaluated the field performance of two carbon-amended models, one proprietary and one non-proprietary, installed at 14 neighboring homes. Influent, effluent, and flow were monitored with high frequency to evaluate changes in nutrient concentrations (N, P) and loads. Site-specific observations and analytical results were regularly communicated among project partners and stakeholders. The process and findings of this intensive evaluation can inform future efforts to reduce excess nutrients in decentralized wastewater and protect water resources.

## 2. Methods

### 2.1. Site selection

The Three Bays watershed (Barnstable, MA) has more than 5000 OWTS, mainly septic systems and a lesser number of cesspools, at an average density of approximately 0.8 per acre (or about 13 per 16 acres). The watershed has an estuarine nitrogen TMDL calling for a 60% N load reduction, with lower and higher targets in subwatersheds (Town of Barnstable, 2020). The Town of Barnstable is engaged in the first of a three-phase, 30-year sewer expansion effort designed to meet this goal; much of the watershed will not be addressed until the second or third phases. The Barnstable Clean Water Coalition (BCWC), a local non-governmental organization, implements alternative engineered and nature-based interventions to reduce current nutrient loading and legacy pollution that source control cannot mitigate. The convergence of partners focused on advancing solutions to this problem determined watershed selection.

Project partners screened the watershed to identify candidate areas for intensive conversion of existing OWTS and assessment, including technologic performance and impact on groundwater quality in a concurrent study. Selection criteria included high wastewater load density, based on data from the Cape Cod Watershed Multi-variant Planner (MVP) tool (available at: watershedmvp.org) and short groundwater travel time to a surface water receptor and non-divergent flow, per an existing regional groundwater flow model (McCobb and Walter, 2019). These criteria served to make sampling more efficient and increase the likelihood of observing a significant change in groundwater. An additional screening criterion was long or indefinite time to sewer availability (20 years or more, per the town’s Comprehensive Wastewater Management Plan, or CWMP, as of 2019), to avoid the potential for competing or repetitive wastewater infrastructure upgrades on the same property in a short time period.

**Fig. 1.**
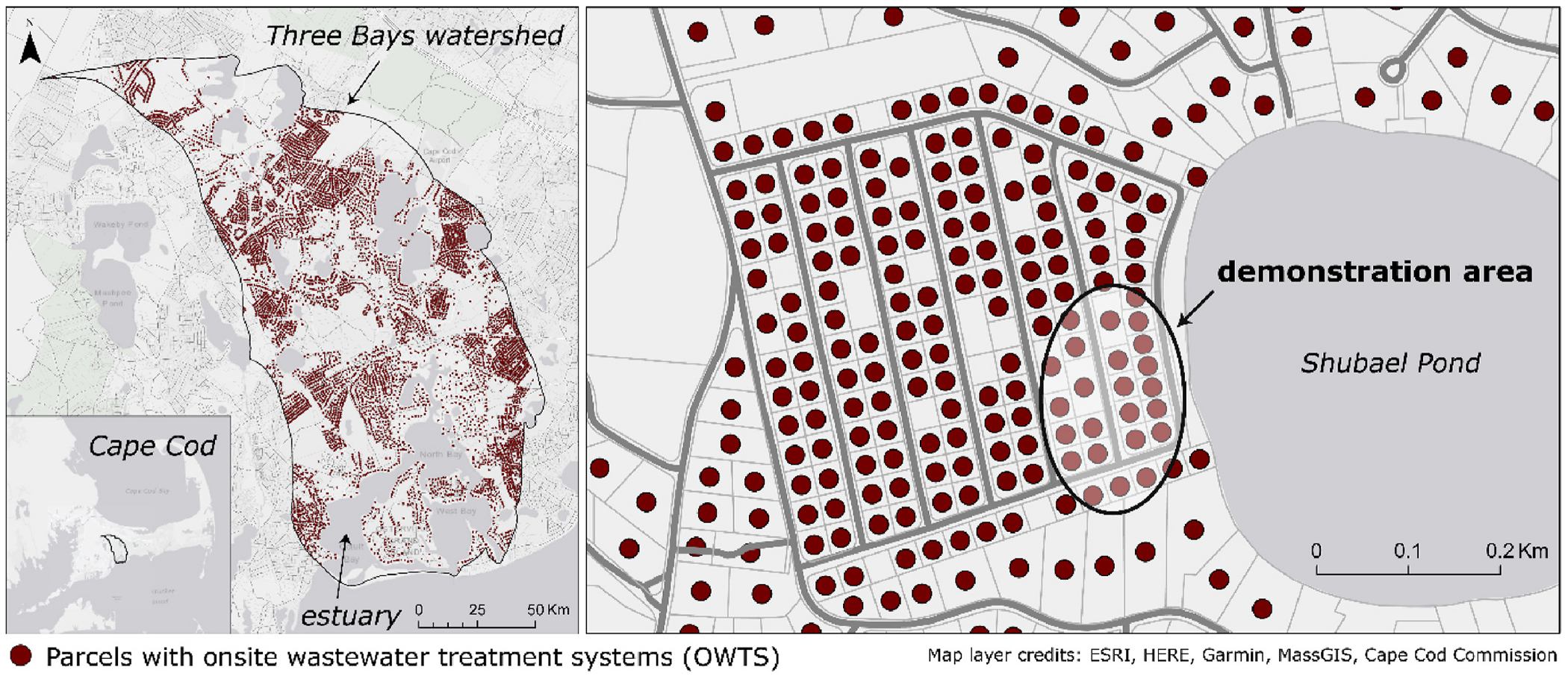
*Left*. The Three Bays watershed in Barnstable, MA. Dots indicate parcels with onsite wastewater treatment systems (OWTS). *Right*. The neighborhood and demonstration area (circled) where the enhanced septic systems were installed.

Candidate areas identified through the watershed screening process were visited by project partners to better assess conditions for OWTS replacements. Considerations included lot layouts and accessibility, with regular spacing and public roads preferred to alternatives. A neighborhood with high housing density (2–4 per acre) on the western shore of Shubael Pond additionally had a homeowners’ association with members and leadership interested in participating in the study. A network of monitoring wells was installed to map hydraulic gradients and sample groundwater water quality. Findings indicated significant wastewater inputs mixed with other sources of recharge, including pond water entering through its western shore and flowing to the southwest. In the southeast of the neighborhood (Fig. 1), a group of contiguous properties was selected such that the upgradient pond could block other septic system sources from confounding monitoring well results. Residents in this cluster of homes were offered full subsidies by BCWC, whose staff coordinated the permitting, engineering, and installation processes.

### 2.2. Septic system replacement and monitoring

**Fig. 2.**
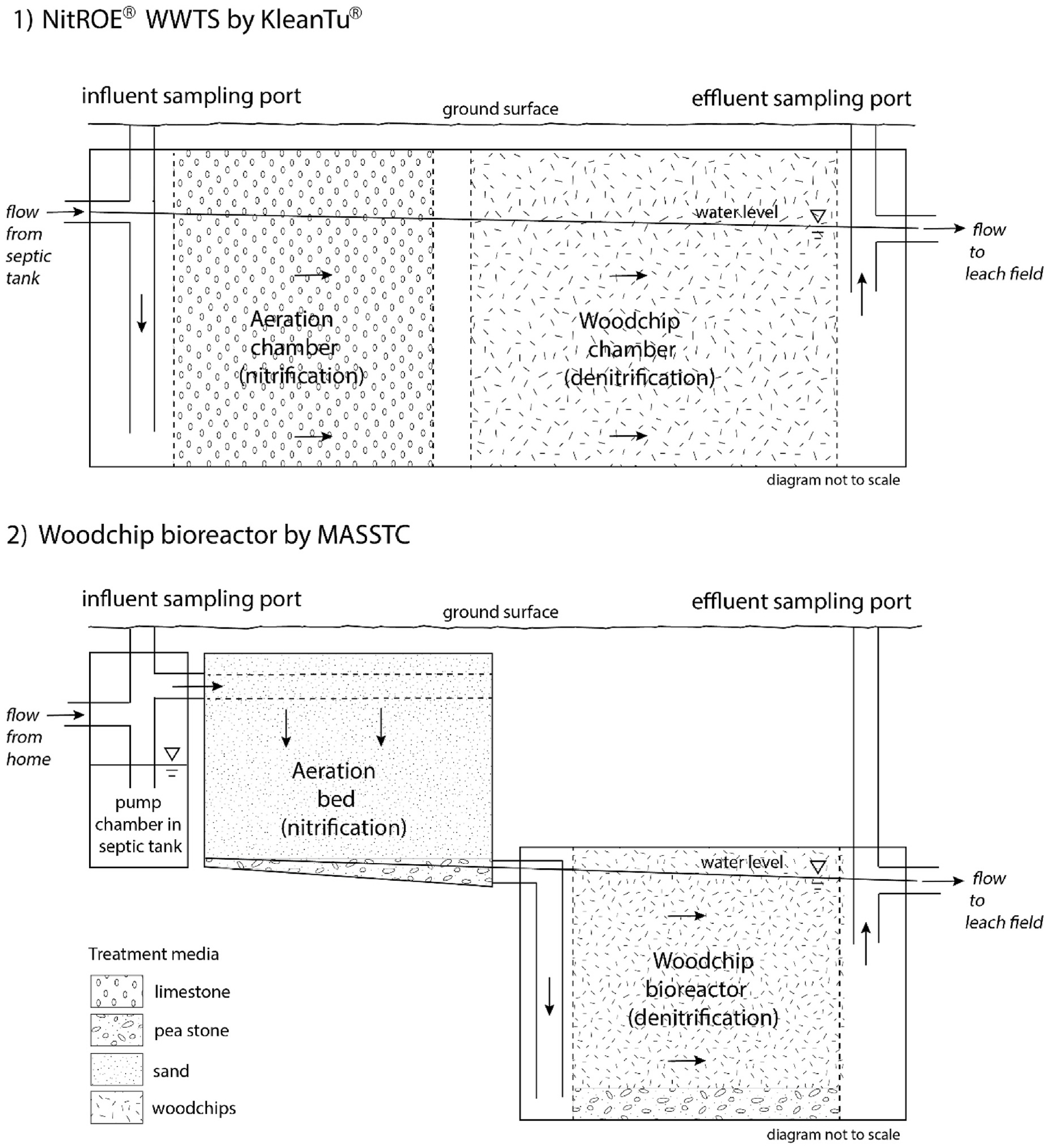
Conceptual diagrams of the septic system enhancements and sampling ports. General flow directions are indicated with arrows. The external air pump and recirculation lines in the NitROE^®^ Wastewater Treatment System (WWTS) tank, which aerate and transfer wastewater from effluent to influent sampling port subcompartments, are not shown for visual clarity. Diagrams are simplified to emphasize the sequence of treatment stages and major system components. Note that they are not drawn to scale. MASSTC = Massachusetts Alternative Septic System Test Center.

Installations occurred over a three-year period (2021–2023) at 14 adjacent single family residential properties. Two technologies were selected by project partners based on demonstrated N removal in limited installations, relative design simplicity, and availability: the NitROE^®^ Wastewater Treatment System (NitROE^®^ WWTS 2 KS), by KleanTu^®^ LLC and a non-proprietary model designed at MASSTC, a testing and research facility for enhanced technologies that is part of the Barnstable County government. Both designs have an aerated stage followed by a woodchip-filled box, or bioreactor. The configuration of the two stages differs between technologies. Conceptual diagrams for each are shown in Fig. 2, emphasizing the general flow paths for wastewater, sampling ports, treatment processes and media.

The NitROE^®^ WWTS is a largely self-contained unit that can be added to an existing septic system, if site conditions allow, or installed as part of a fully new system. The NitROE^®^ consists of a 1500 or 2000-gallon concrete tank with two primary treatment compartments. The first compartment, filled with limestone rocks, is continuously aerated with an external pump located at the land surface and provides nitrification and alkalinity control. The second compartment is filled with woodchips to support denitrification under anoxic conditions. Recirculation from the outflow to a small antechamber preceding the first compartment allows for additional treatment opportunities and is facilitated by the same air pump in an otherwise gravity-driven system.

The second design by MASSTC further separates the nitrification and denitrification stages of treatment. Aeration occurs in a lined sand bed that is dosed with septic tank effluent by a low pressure-pump housed in a chamber of the septic tank. Dispersal throughout the bed is facilitated by a GeoMat^™^ 3900, a modular drainfield system consisting of geotextile fabric and perforated pipe. Percolating effluent in the aerobic 18” sand bed is nitrified and collected in an underdrain. The bed, filled with C-33 washed concrete grade sand, measures 35 by 14 ft (length by width) and is 1.5 ft thick. It is underlain by a sloping layer of pea stone and polyethylene liner. The bed was amended with alkalinity by adding 10 pounds of sodium bicarbonate to the pump chamber postinstallation. Gravity moves drainage from the sand bed to the bottom of a 1500-gallon woodchip bioreactor, where denitrification occurs, and on to final effluent disposal in a second leach field.

Actual engineering designs and details varied among sites according to the locations, elevations and condition of existing OWTS components. A total of 13 NitROE^®^ units were installed at sites A-L, as they are identified in graphics below, with 2 as part of full new septic systems (sites A, B) and 11 (C-L) as retrofits between an existing septic tank and/or leach field. Site K and N received the 1500-gallon NitROE^®^; others have a 2000-gallon unit. The remaining site M received a complete new septic system designed by MASSTC. The imbalance in type of systems installed stems from funding and site constraints; the non-proprietary system, with two leach fields, has a larger spatial footprint. Half of the 14 sites needed new leach fields, made with 500-gallon concrete chambers, to replace cesspools or otherwise achieve regulatory compliance. Pan lysimeters were installed below 5 of the new leach fields, and within the single sand bed, to collect additional diagnostic information and final effluent. Each design was approved by the local Board of Health.

Installations proceeded at the pace of approvals, available funding, and homeowner readiness. Private funds were raised by BCWC to install all proprietary systems. The non-proprietary system was installed with partial funding from a $20,000 grant to Barnstable County, specifically MASSTC, from EPA’s Southeast New England Coastal Watershed Restoration Program (SNEP) for the project “Full Scale Assessment of Non-Proprietary Passive Nitrogen Removing Septic Systems” (grant ID: 00A00249). In addition to the septic system modification or full replacement, installed exterior to the home, a Neptune T-10^®^ residential water meter was placed within the interior plumbing at a location suitable for recording indoor flows (outdoor usage does not flow through the septic system enhancements). Site E had no suitable location in its plumbing layout. Following installation and start-up the new system components were seeded with activated sludge to hasten the growth of microbial populations.

Monitoring by MASSTC commenced two months post-installation. Samples were collected monthly from the septic tank outflow (“influent” to the N-reducing unit), from the outflow of the system enhancement (“effluent”, from either the NitROE^®^ unit or woodchip bioreactor) and from the pan lysimeters (leach field percolate). Influent and effluent sampling ports are labeled in Fig. 2. Port covers at the land surface were uncovered and fluids accessed with a 12 V submersible Whale^®^ pump, flushed and drained with clean water between samples to limit cross contamination. Field measurements of temperature, specific conductance (SpC), turbidity, pH, and dissolved oxygen (DO) were made onsite at the time of sample collection with a YSI ProDSS^™^ multimeter, calibrated daily for SpC, pH, DO and turbidity per MASSTC standard operating procedures. Laboratory samples were collected in plastic bottles, field acidified as needed with sulfuric acid, and transported on ice to the Barnstable County Water Quality Laboratory.

Analytical parameters and measurement frequency followed permit requirements and demonstration objectives. Samples were analyzed quarterly for 5-day biochemical oxygen demand (BOD_5_), total suspended solids (TSS), alkalinity, and ammonia + ammonium, reported as (NH_3_). Nitrite (NO_2_^−^), nitrate (NO_3_^−^), total Kjeldahl nitrogen (TKN, which includes organic N, ammonia + ammonium), were analyzed monthly, and total nitrogen (TN) was determined as the sum of the three components. Total phosphorus (TP) was also analyzed monthly, in influent and effluent samples only, as it was not the focus of the designs or evaluation but is important to local stakeholders for management of freshwater bodies. Nutrients were analyzed on a more frequent basis to better evaluate performance and to inform system adjustments. Analytical methods, reporting limits and sample handling are specified in Table 1.

Wastewater flow readings were recorded at hourly intervals and downloaded during quarterly system inspections. Inspections followed the operations and maintenance (O&M) procedures outlined in the Massachusetts Department of Environmental Protection (MassDEP) I/A septic system and manufacturer checklists. Included are visual and olfactory assessments, checks for equipment failures, ground surface issues, and sludge levels. Air supply line pressures were also checked, recirculation rates adjusted to ensure flow, alarms and pump cycle counts recorded as appropriate for the design. The sand bed laterals were flushed during one visit and pressures checked to ensure even wastewater distribution. MASSTC’s O&M team was in regular contact with the NitROE^®^ manufacturer throughout sampling and inspection efforts. In some cases, inspections facilitated direct communication with residents.

Field inspection notes are provided along with field and laboratory methods, reporting limits, and values in version 2 of the published dataset (Wigginton et al., 2023, available at: https://doi.org/10.23719/1529539). Data from this work are also stored in Barnstable County’s I/A septic system monitoring and compliance database. Statistical summaries for the systems in this study can be compared with installations of the same and other technologies at septic.barnstablecountyhealth.org.

### 2.3. Performance assessment

Performance of the enhanced I/A septic systems was reviewed by project partners on a continuous basis during the monitoring period. Data are summarized here for the 13 systems with at least a full year of monitoring data, by site, port and parameter assessed. Two key outcomes include changes in 1) nutrient concentrations and 2) total loads in influent and effluent of the N-reducing enhancement. A third summary outcome presented is the reliability of systems with respect to meeting the effluent performance goal of 10 mg/L TN.

Mass loads of nutrients were estimated in self-consistent units of mass (M), length (L) and time (T) as follows:

$$load\left[\frac{M}{T}\right]=concentration\left[\frac{M}{L^{3}}\right]\times flowtosystem\left[\frac{L^{3}}{T}\right]$$

Loading rates were calculated for each system from its mean influent and effluent concentrations and flow rates during the monitoring period, a conservative estimate that may overestimate loading when the data for a system are positively skewed. Results are expressed in units of kilograms per year (kg/yr) for consistency with other reports and TMDLs.

The statistical significance of changes in concentrations and loads was evaluated using Wilcoxon signed ranks tests on paired samples, using R version 4.1.3 (R Core Team, 2022) and package *rstatix* 0.7.2 (Kassambara, 2023). Test sample values were the paired long-term means for the given outcome by system, port, and parameter. Comparisons were conducted for two time periods: 1) the full monitoring record and 2) a 12-month subset, period of overlap among systems that includes only the most recent data (2023). The full record is potentially confounded by variable sample numbers and time spans due to asynchronous installation dates. The shorter period could potentially be confounded by start-up effects for systems installed closer to the beginning of the period, though no such effects are evident in the data.

Reliability of each system, defined here as the percentage of the time each could be expected to achieve the effluent quality goal under comparable operating conditions, was estimated in two ways. The first is the percentage of total effluent samples, by system, with values below 10 mg/L. The second probabilistic approach follows Oakley et al. (2010), after Niku et al. (1979), and penalizes reliability scores according to system variability, but it assumes a lognormal distribution to which these data do not conform. The two may be considered high and low estimates, respectively, for systems with greater temporal variability. In cases of low variability in effluent quality, the reliability estimates are nearly the same.

## 3. Results

Results are provided for the first 13 systems that were installed in 2021 and 2022. This means that monitoring data for each system spans at least one full year (2023) and accounts for seasonal variability in temperature that can affect biologically mediated performance.

### 3.1. Water use and wastewater flow

**Fig. 3.**
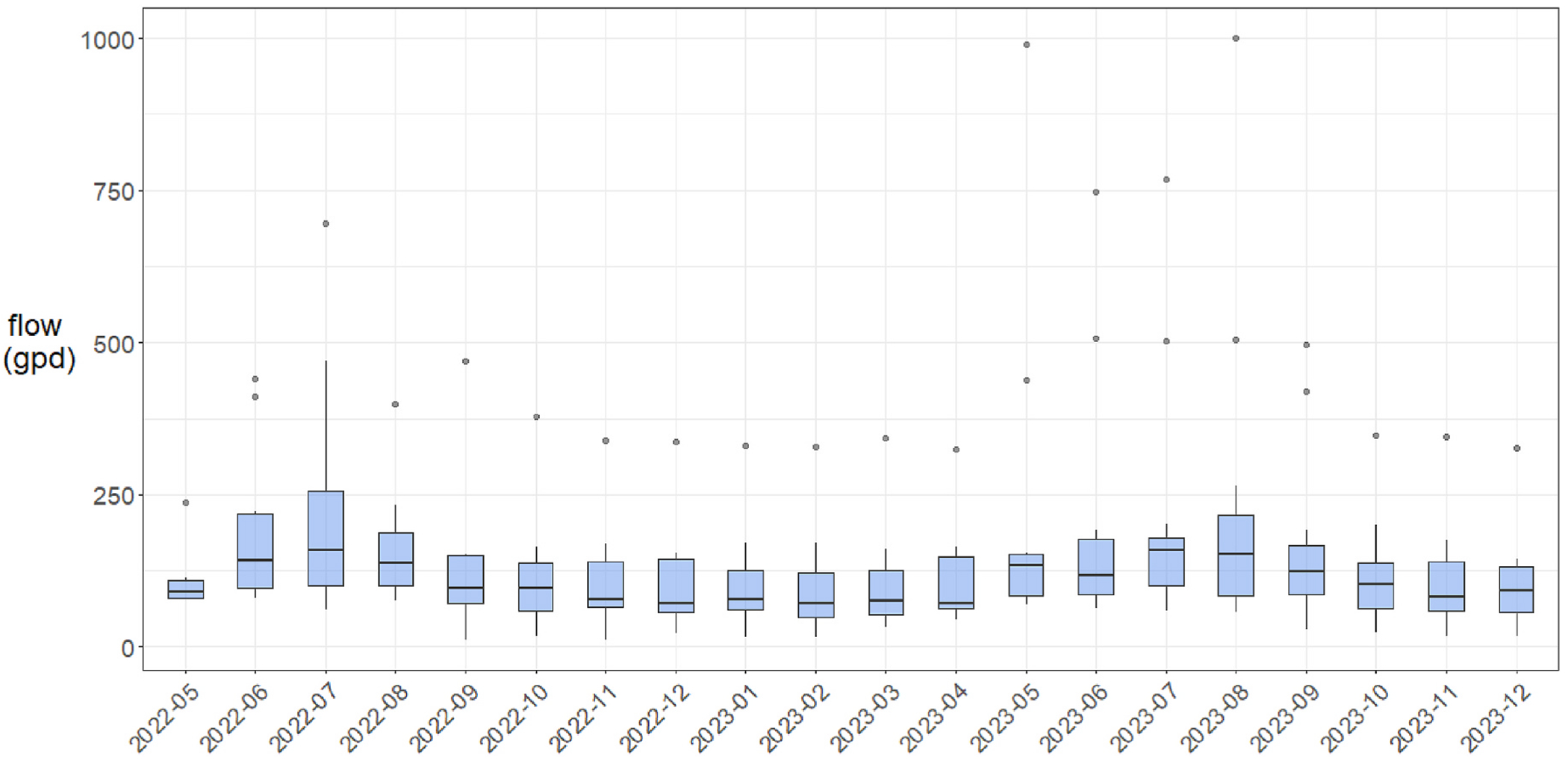
Mean daily flow (gallons per day) by month in the monitoring period. Measurements capture indoor water use that is discharged to 13 Innovative/Alternative septic systems in Barnstable (Cape Cod), Massachusetts, a cohort comprised of two models that incorporate woodchip bioreactors to enhance N removal. Box plots depict the minimum, first quartile, median, third quartile, and maximum flow rate among sites, with outliers depicted as single points. Note that flow data collection began at different times and thus some boxes do not contain data for all sites. All sites with flow meters (n = 12) are represented from 2022-09 onward.

Metered flow to the systems was highly variable among sites and over time. Indoor water use ranged from 0 to 2098 gallons per day (gpd). The mean daily flow was more moderated; 61–390 gpd, or 149 gpd on average across sites. Flow records indicate that residences were, for the most part, occupied year-round. Use rates were slightly higher during summer months (Fig. 3). Periods of no flow lasted less than two weeks, though a full month of very little flow (<10 gallons) was recorded at one site. At another, excessive flow was used to detect and fix a leaky toilet. Daily flow rates often exceeded the design specification of 330 gpd for MASSTC’s non-proprietary system (median: 369 gpd; mean: 390 gpd). Samples from a lysimeter in the sand bed indicated saturation, low DO, and diminished nitrification.

Flow rates co-determine the hydraulic retention time (HRT) for water in a reactor, along with its volume and internal flow paths. Bulk estimates of HRT in the N-reducing system enhancement were made using mean daily flow rates (Table 2), unit volumes (1500 or 2000 gallon) and an assumed 50% void space. By this approach, estimates of bulk HRT range from 1.9 to 14.9 days. The mean HRT estimate for NitROE^®^ units is 9.4 days. For MASSTC’s non-proprietary system the HRT of the woodchip bioreactor was estimated to be 1.9 days, given the small unit volume and high water use at the site. Importantly, bulk HRT estimates do not account for a distribution of residence times caused by short-circuits, stagnation zones, and dispersion in the porous matrix of the woodchip reactor.

### 3.2. Nutrient concentrations and loads

**Fig. 4.**
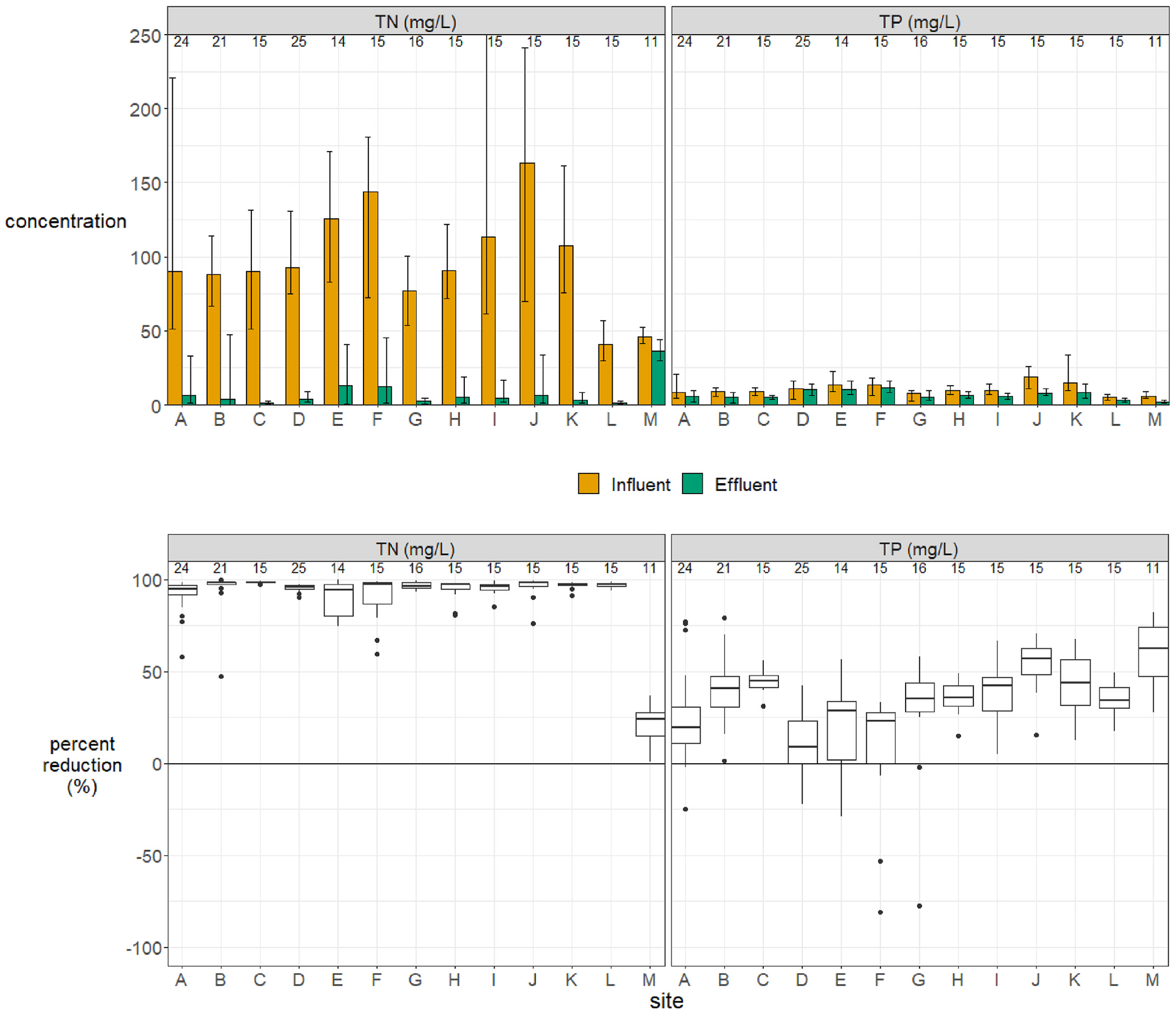
*Top*. Nutrient concentrations—total nitrogen (TN) and total phosphorus (TP)— by site and sampling port. The mean and range of values are shown for wastewater from 13 Innovative/Alternative septic systems in Barnstable (Cape Cod), Massachusetts, a cohort comprised of two models that incorporate woodchip bioreactors to enhance N removal. The sites are described in section 2.1. The number of samples per site is indicated above each bar. One influent TN value of 420.8 mg/L at site I is out of the plotted range. *Bottom*. Percent reduction of nutrients from influent to effluent, by site. Variability over time and among sites is indicated with box plots. Box plots depict the minimum, first quartile, median, third quartile, and maximum, with outliers depicted as single points.

Mean influent TN concentrations were 40.8–163 mg/L among sites, or 97.8 mg/L on average for the group. The mean influent TP concentration was 10.6 with a range of 5.0–18.7 mg/L among sites. In treated effluent, mean TN concentrations ranged from 1.4 to 36.6 mg/L among sites, with a group mean of 9.8 mg/L (Fig. 4). Excluding site “M”, where N removal was known to be compromised due to excessive water use, the group mean for the NitROE^®^ systems effluent TN was 7.6 mg/L. Effluent TP ranged from 2.2 to 12.1 mg/L with a mean of 6.9 mg/L. Median values are lower: half of systems had long-term average effluent TN < 6.2 mg/L and TP < 5.8 mg/L. The five lysimeters showed a mean long-term TN of 6.1 mg/L and no significant additional reduction relative to paired effluent samples. Differences between effluent and lysimeter samples are shown in the supplementary materials.

Nutrient concentrations reductions were high for most systems when operated as designed. TN concentrations were reduced from influent to effluent by a long-term average of 89.9–98.5%, averaging 94.9% (median: 95.6%) in the 12 NitROE^®^ units (Fig. 4). Site “M” achieved a modest average removal rate of 20.6% (median: 24.3%) despite operational issues. Reduction in TP concentrations by these systems averaged 30.1% (4.8–53.6%; median: 35.4%), apparently due to incidental sorption and/or precipitation, as they were not designed for P removal. A few sampling events indicated higher concentrations in effluent than influent, but net reduction was observed at all sites over the long-term.

**Fig. 5.**
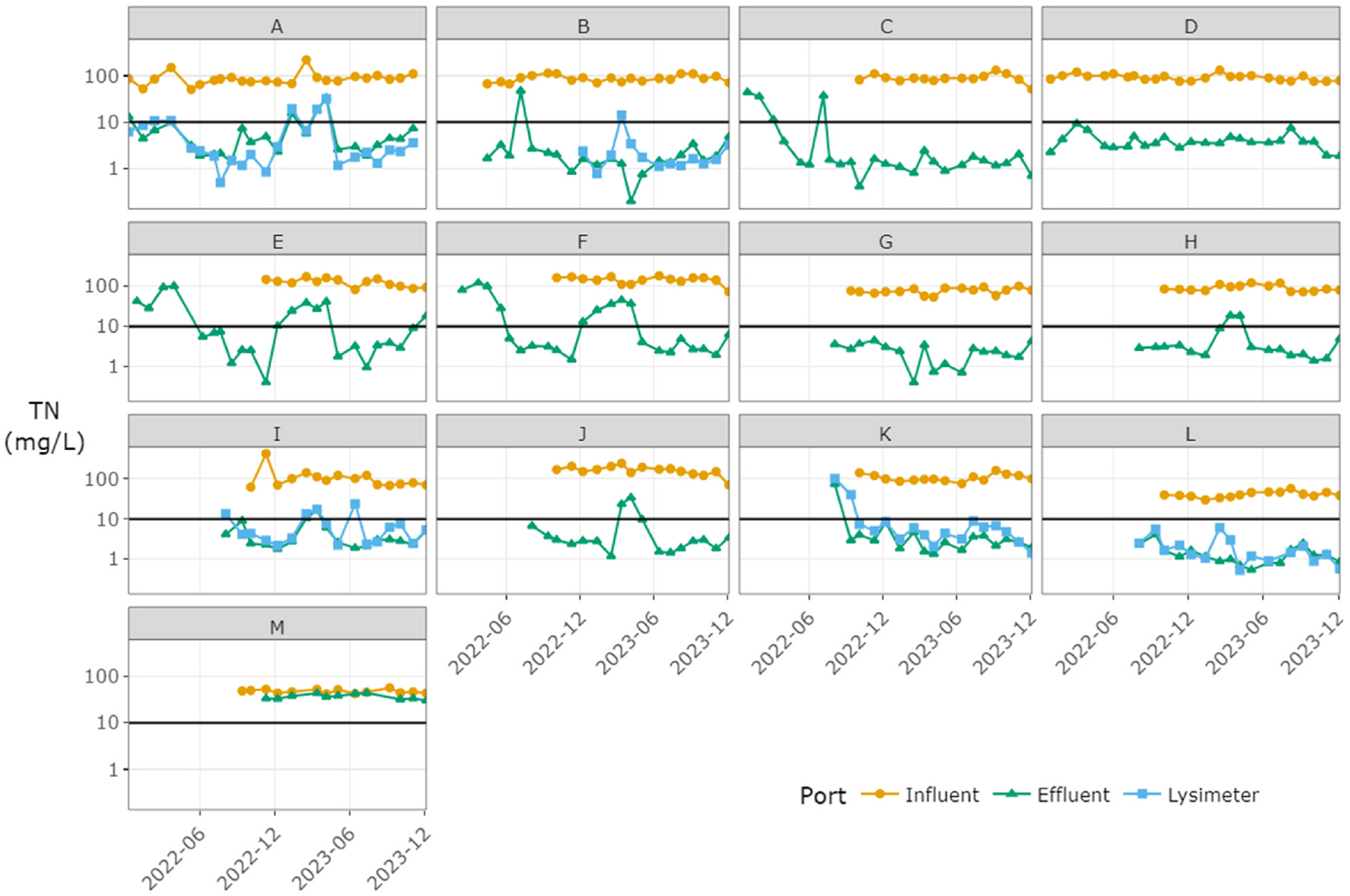
Total nitrogen (TN) concentrations (mg/L) over time, by site and sampling port, in wastewater from 13 Innovative/Alternative septic systems in Barnstable (Cape Cod), Massachusetts, a cohort comprised of two models that incorporate woodchip bioreactors to enhance N removal. Note that the vertical axes have a logarithmic scale. The performance goal of 10 mg/L is indicated with a black line.

Performance showed different patterns among sites and over time unrelated to influent concentrations (Fig. 5.). Three systems met the goal of effluent TN < 10 mg/L from first sampling and consistently thereafter (sites D, G, L). Eight systems met the goal in early sampling, but four of these had intermittent 1–2 month periods of elevated effluent levels, up to 47 mg/L TN, at a later time (sites A, B, H, I). Four systems showed an initial lag in performance of 4 months or less linked to insufficient nitrification (sites C, E, F, K). In most cases, goal attainment was not dependent on season, however, four systems showed diminished denitrification during colder months, with effluent TN in excess of 40 mg/L in some cases (sites E, F, H, J). Single month spikes occurred at two sites in the summer, preceded and followed by multiple months of low effluent TN (sites B, C). The ancillary water quality and flow data collected were not always sufficient to discern the causes of performance variability within or among systems. Likely there are unmeasured factors that could explain some of this variability (see Discussion). Additional presentation of the ancillary data is provided in the supplementary materials.

**Fig. 6.**
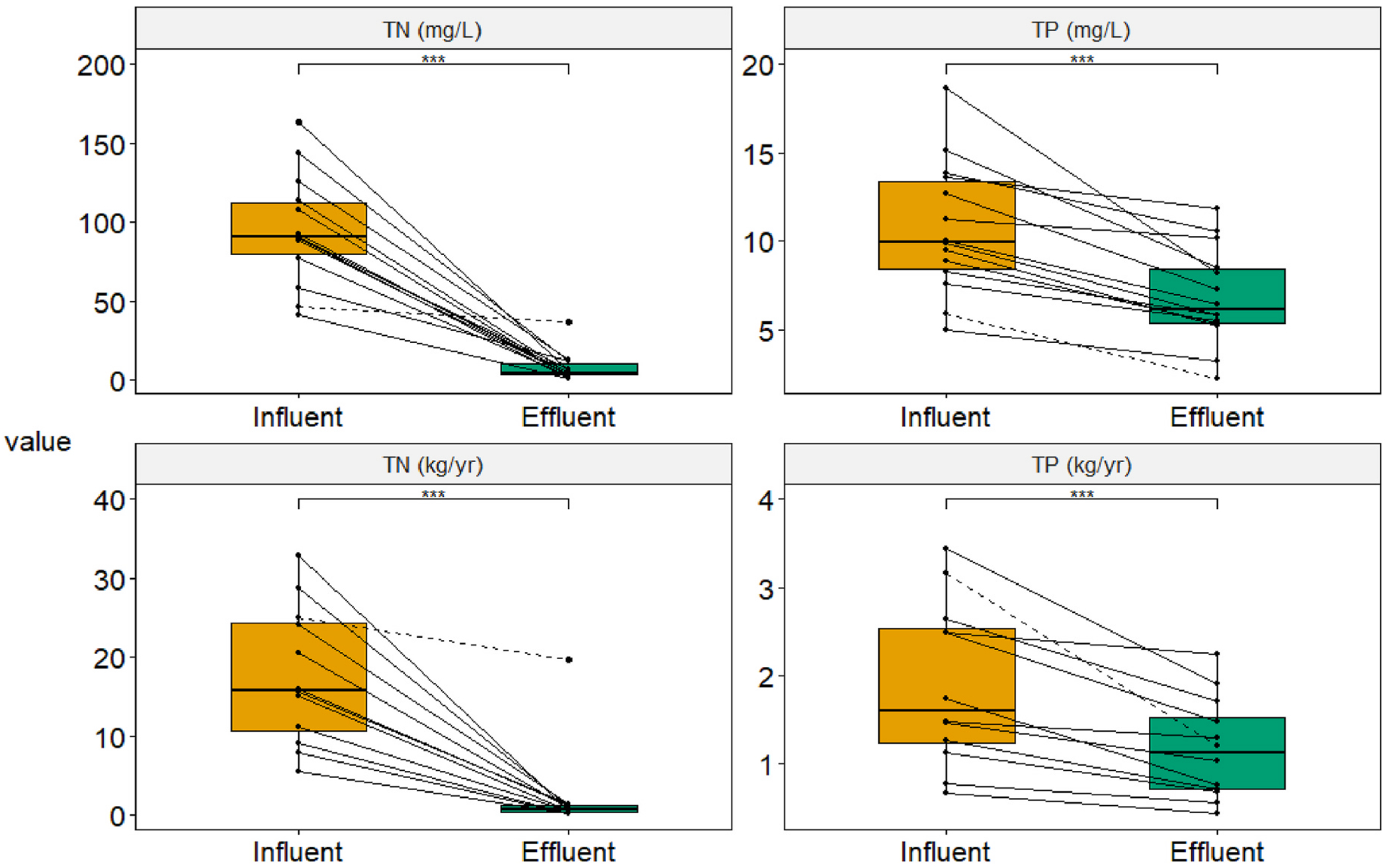
Nutrient concentrations (*top*) and estimated loads (*bottom*) of total nitrogen (TN) and total phosphorus (TP) in the wastewater of 12 Innovative/Alternative septic systems installed in Barnstable (Cape Cod), Massachusetts. The cohort is comprised of two models that incorporate woodchip bioreactors to enhance N removal. Only sites with flow meters and data, needed for load estimates, are presented for consistency within the figure. Each point is a mean value for the full monitoring record and sampling port, with lines connecting paired values for each system. The dashed lines correspond to the non-proprietary system. Note the differences in y-scales between plots. The statistical significance of differences between influent and effluent values for the group is indicated above the brackets (***, p < 0.001). Box plots depict the minimum, first quartile, median, third quartile, and maximum, with outliers depicted as single points.

Load reductions, which are mediated by flow rates, generally followed the concentration outcomes (Fig. 6). Influent TN load estimates ranged from 5.5 to 34.2 kg/yr, averaging 17.5 kg/yr among all sites with water meters (n = 12). In effluent the estimates ranged from 0.16 to 19.9 kg/yr (mean: 2.3 kg/yr). Using median values, to reduce the influence of outliers, TN loads were reduced from an estimated 15.9 to 0.72 kg/yr, or 95.5% from influent to effluent. Influent TP load ranged from 0.6 to 3.6 kg/yr, averaging 1.9 kg/yr, The median TP load reduction was from 1.6 kg/yr to 1.2 kg/yr, or 25%. Nutrient removal by these enhanced treatment units was highly significant (p < 0.001; Fig. 6.). Additional removal between paired effluent and lysimeter samples was not significant, as shown in the supporting materials. All systems showed net removal of both nutrients, as indicated by the line slopes, in terms of concentration and mass load.

Comparisons made on the full dataset may be confounded by the unbalanced sample sizes among systems due to asynchronous installation dates and corresponding discrepancies in seasonal or other temporal factors. Reanalysis with a balanced sample set restricted to the most recent 12-month period in the data collection (2023) showed no meaningful changes in plotted outcomes or differences in significance levels for the same comparisons, as is shown in the supporting materials. Results are therefore based on the full dataset, which includes greater variability for some systems.

### 3.3. Ancillary water quality measures

Field parameter values were within expected ranges. Sample temperatures oscillated between 5.3 and 26.1 °C from winter to summer (see Table 3.). Dissolved oxygen (DO) typically ranged from 5 to 12 mg/L in treated effluent samples and varied inversely with temperature, consistent with higher microbial activity in the enhanced treatment units during warmer months. No clear temporal trends were observed in other field parameters. pH was near neutral and within the acceptable range (6–9) for most samples. Influent samples tended to be slightly acidic (median: 6.8) while effluent and lysimeter samples tended to be slightly basic (median: 7.4). Specific conductance was generally 400–800 μS/cm, with influents on the high and effluents on the low end of the range.

Laboratory values for ancillary water quality measures were generally within acceptable ranges. Effluent TSS values ranged from non-detectable to 180 mg/L (mean/median: 23.8/7.8 mg/L). Effluent BOD_5_ ranged from non-detectable to 130 mg/L (mean/median: 12.1/1 mg/L). Values indicate high carbon reduction, secondary treatment, and compliance with regulatory limits (30 mg/L for both parameters) on most occasions (>80% of samples). Elevated values showed no temporal trends but rather occurred episodically. Alkalinity in influent ranged from 140 to 660 mg/L as CaCO_3_ (median: 340 mg/L). Effluent alkalinity ranged from below detection to 520 mg/L as CaCO_3_ (mean/median: 115/110 mg/L). Values for all water quality data are summarized by port and parameter in Table 3.

### 3.4. Reliability

**Fig. 7.**
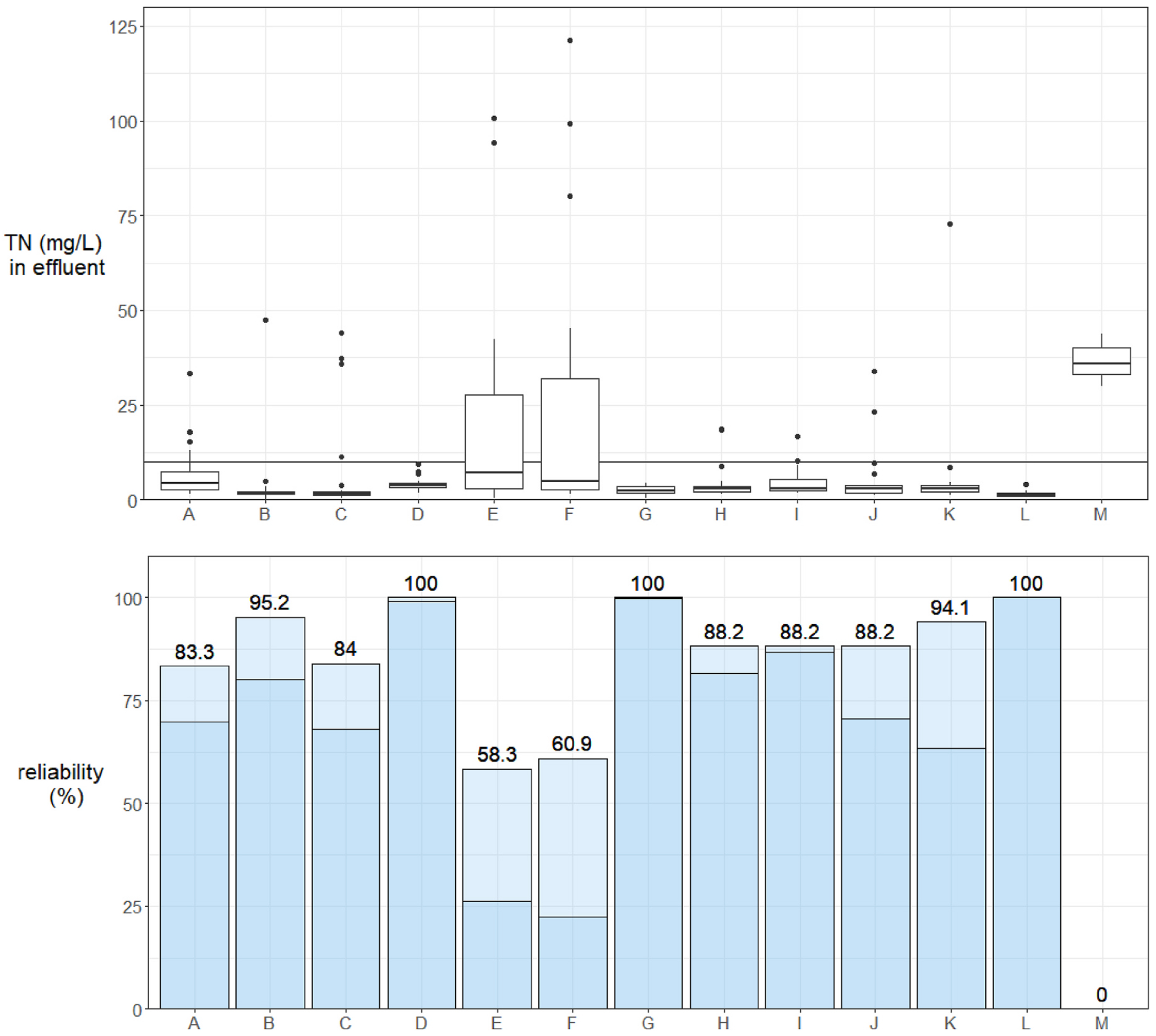
*Top*. Total nitrogen (TN) in wastewater effluent from 13 Innovative/Alternative septic systems in Barnstable (Cape Cod), Massachusetts, a cohort comprised of two models that incorporate woodchip bioreactors to enhance N removal; data are presented site, as described in section 2.1. The performance goal of 10 mg/L is indicated with a black line behind box plots. Box plots depict the minimum, first quartile, median, third quartile, and maximum, with outliers depicted as single points. *Bottom*. Reliability of systems for meeting the goal estimated two ways. Labeled values are the high estimate, or the absolute percent of samples that met the goal during the study period. Darker bar heights indicate the lower probabilistic estimates.

Reliability with respect to meeting the performance goal of 10 mg/L TN in effluent was variable, but high for the group as a whole. The median reliability score for the group of systems was 70.6–88.2%, depending on method. Fig. 7 uses box plots (top) to show the variability in effluent TN alongside the performance goal to provide visual context for reliability estimates (bottom). The non-proprietary system at site M never achieved the performance goal during this period owing to operational issues; its reliability score is currently 0 in both cases, though full performance potential under design-compatible conditions is not captured in this work. Among NitROE^®^ systems, the lower, probabilistic reliability estimates ranged from 22.5 to 99.9% (mean: 72.2, median: 75.3%). The more intuitive, strictly event-based reliability estimates ranged from 58.3 to 100% (mean: 86.7, median: 88.2%).

## 4. Discussion

The enhanced Innovative/Alternative (I/A) septic systems in this field demonstration were highly effective at removing nitrogen (N) from residential wastewater. Technologies included proprietary and non-proprietary models, both amended with a woodchip bioreactor to boost denitrification. Effluent concentrations were typically less than the 10 mg/L total nitrogen (TN) performance goal, and in many cases below 5 mg/L, a significant improvement over most available alternatives. Mean effluent TN among systems was 7.6 mg/L (median: 6.2 mg/L) when operated consistently with their design. Influent TN concentrations and loads were reduced by more than 90%, on average, across the group, compared with removal rates of 10–40% for a conventional septic system or bottomless sand filter (Costa et al., 2002; USEPA, 2002; Rich, 2008). Several of the systems performed well from start-up and consistently thereafter. Others showed a drop-off in N removal during cold periods, or a brief spike during the warm season. Some needed physical adjustments following start-up to establish high performance. Most systems showed high reliability (>80%) with respect to meeting the performance goal.

Departures from design involved compromised aeration and recirculation, excessive hydraulic loading, or a combination of the two. In systems aerated with an external pump (i.e., the NitROE^®^ units at sites A-L), performance declined when air lines leaked and would similarly drop if the pump was not on. At site M, where percolation in a sand bed was used to aerate and nitrify effluent, high water use led to over saturation. Waterlogging may also have caused overgrowth of the biomat, limiting infiltration. The system at site M was operated at a hydraulic loading rate that exceeded its design specification. The assessment of this non-proprietary option therefore does not indicate its full performance capacity but highlights the need for self-consistent use conditions. Water use records for a proposed location may be useful where available. Excessive flow also reduces hydraulic retention time, potentially undermining treatment in either system. It may be possible to reduce or redistribute water use over time within a household through changes in fixtures or habits. Both systems enclose the woodchips in a tank with access ports, which forces all flow through the anoxic bioreactor and allows for the media to be replaced if needed.

Performance was often, but not always, clearly related to operating conditions and the diagnostic measures that were made aside from TN. Periods of inadequate aeration, for example, corresponded with high TKN in effluent. For other transient outcomes there is no definitive explanation in the data collected. Elevated NO_3_^−^ for brief periods (<2 months), amid otherwise low TN, indicates reduced denitrification due to factors not resolved by inspection notes or ancillary physicochemical parameters. The observed cold weather performance, diminished in some but not most systems, is inconsistent with other lab and field studies and rates of microbial activity, which are generally understood to rise and fall with temperature, doubling for every 10 °C (Amador and Loomis, 2020). The minimum effluent temperature in this work was only 5.3 °C and potentially not limiting to treatment in systems where other necessary conditions were met, for example the degree of microbial community establishment, which may explain observed differences in seasonal performance; beginning winter with a diverse and robust community may have helped some systems sustain low effluent TN through it.

Unexplained discrepancies in treatment may also be related to differences in hydraulic efficiency among systems, or in the use of inhibitory household inputs. The volume, chemical, and biological composition of residential wastewater can vary greatly, a fundamental design challenge for onsite treatment that likely contributes to underperformance of advanced systems in field settings (Oakley et al., 2010; Amador and Loomis, 2020). Systems in this study experienced a high range of daily and annual average flow rates and may have received, on a recurring or episodic basis, harsh cleaning products, antibiotics, chemotherapies, or others inputs lethal to microorganisms. The community composition in other denitrifying septic systems varied with design, treatment stage, and season (Langlois et al., 2020; Ross et al., 2020b). Biological processes more broadly, including dissolution of the woodchips by cellulolytic bacteria, depend in complex ways on microbial diversity and competition, availability of dissolved oxygen and alkalinity, pH, temperature, flow paths, media-water contact and residence times. Some of these factors are not well controlled in field settings and impractical to monitor. Uncontrolled factors are implicitly represented here in reliability scores that are more informative when applied to more installations and use cases. Seasonal occupancy, for example, not assessed here since homes were mostly occupied year-round.

This demonstration did not include a control group, but prior field efforts suggest how efficiently septic systems with woodchip bioreactors remove N relative to alternatives. The La Pine National Demonstration Project, conducted in Oregon in the early 2000s, tested 11 technologies with varying process configurations. Results showed superior N-removal with this approach and indicated that achieving TN below 10 mg/L in effluent consistently could require a secondary carbon source (Rich, 2008). More recently, 5 advanced technologies that rely on recirculation, without supplemental carbon, have been systematically monitored over 1–3 years at homes in Rhode Island. Median effluent TN ranged from 11.3 to 33.8 mg/L among technologies, with values typically in the low to mid-teens (Lancellotti et al., 2017; Ross et al., 2020a). Similar effluent quality is observed for I/A systems installed in Barnstable County, Massachusetts, where compliance data represent a larger set of technologies (Martin and Johnson, 2020; BCDHE, 2024). In Suffolk County, New York, the same non-proprietary boxed design used in this demonstration outperformed configurations with woodchips layered in lined and unlined leach fields, for a mean effluent TN of 5.3 mg/L and 94% removal under field conditions (Gobler et al., 2021). The three regions have similar climate and populations that make data from them most directly comparable to this work. A more complete comparison of treatment efficiency would consider flow and influent concentrations that are often not collected in field settings.

Sustaining high performance by enhanced septic systems, as with other kinds of advanced technologies, requires additional involvement by a specialized public or private entity. Homeowners are typically responsible for maintenance of conventional septic systems, which not infrequently experience preventable hydraulic failures that can be disruptive, expensive, and cause for exposure to pathogens. Alternative septic systems are at greater risk of not achieving intended outcomes owing to additional hardware components, treatment contingencies and less obvious modes of failure (i.e., exceeding effluent quality limits). Tools to help systems perform as designed include O&M contracts, user manuals (reinforced with other modes of communication), visual and auditory cues of system status, and remote monitoring of component function and alarms. Avoidance or moderation of wastewater inputs that clog components (e.g., hair, trash, grease), or that are acutely toxic to microbes are beneficial practices for any septic system. With N-removing models, sludge seeding may accelerate microbial growth and improve performance following installation or disturbance.

Given the additional complexities of enhanced onsite wastewater treatment, localities may benefit from a responsible management entity (RME), essentially a utility for decentralized systems. RMEs can consolidate information about which technologies are best performing and suited to a given location. Beyond effluent quality goals, considerations include capital and recurring costs, existing infrastructure, lot layout, depth to groundwater, availability of backup power and customer service. RMEs can help coordinate system selection, design, installation, O&M, monitoring, schedule inspections and pump-outs. Economies of scale could also be achieved with clustered I/As, where multiple homes connect to an otherwise decentralized treatment system. Load reductions to surface waters may be realized more quickly through coordinated system placement. The individual enhanced I/A septic systems in this demonstration were clustered spatially within a small area, which made site visits more efficient. They formed part of the basis for a Septic Utility Program at the Massachusetts Alternative Septic System Test Center (MASSTC), the first of its kind in the state.

Successful use of enhanced I/A septic systems can benefit from broader knowledge-sharing among municipal, state, federal, and tribal authorities and the public. MASSTC maintains a monitoring and compliance database for systems installed in Barnstable County, including O&M history and effluent sample results for more than 25,000 events (as of 1/2024, see BCDHE, 2024). This information, collected over a longer time period and greater range of technologies and real-world conditions, supports stakeholders in wastewater management decision-making, including where and how to choose among centralized and decentralized alternatives. Data exchange and reciprocity agreements among states could help state regulators vet new OWTS technologies more quickly. A recognized concern, however, with increased availability of enhanced septic systems is that without additional safeguards, which are often under the purview of local jurisdictions, “the inevitable development of sites formerly considered undevelopable” (RIDA, 2016) may undermine or reverse gains in N load reduction and exacerbate other environmental concerns.

Treatment goals for OWTS effluent quality can include pollutants besides N and pathogens. Phosphorus (P) loads were significantly reduced by systems in this study, though effluent remained enriched (>100x) relative to threshold levels for eutrophication in freshwater environments like lakes and ponds (~0.03 mg/L P, per Smith, 1999). Phosphorus is attenuated to a much greater extent than N in the subsurface as it tends to bind to porous media in the leach field of a septic system or underlying unsaturated zone (Robertson et al., 2019, 2020; Wehrmann et al., 2020). Sequestration capacity varies with time and by location, however, and controlling both N and P may be needed to protect a given water body (Conley et al., 2009; Paerl et al. 2016, 2018). Other regulated, and a large class of unregulated contaminants of emerging concern (CECs) found in wastewater have environmental fates and effects that are even less clear (Schaider et al., 2017). Undoubtedly, the total composition and distribution of pollutant loading has complex consequences for human and non-human populations.

Some of this loading can be reduced at the source without an enhanced onsite treatment system or sewer connection via behavioral changes. Choices of personal care and household products, diet, and handling of food wastes affect the quality of wastewater effluent. Most of the nutrients in residential wastewater derive from urine. Urine diversion, food scrap composting, blackwater separation and composting toilets are effective at removing nutrients from effluent to stem eutrophication and provide opportunities to recover them for productive use as fertilizer (see, e.g., Wilsenach and Van Loosdrecht, 2004; Boyer and Saetta, 2019; Moussa et al., 2021). Examples exist throughout the world, driven by local conditions and stacked benefits (Larsen et al., 2013); on its own, recovery of more valuable resources like P and precious metals is still generally not economically viable, even in the setting of municipal wastewater treatment (Mayer et al., 2016) though successful small-scale programs do exist. Incentives could change in the future, however, to favor resource recovery over waste disposal.

Managing wastewater nutrients and co-pollutants where they are discharged is also more effective than remediating them downstream. Ground- and surface waters can be partly treated with interventions like fertigation, permeable reactive barriers, restored wetlands, and shellfish aquaculture, among others being implemented in and beyond Cape Cod. Performance depends on specifics of the approach, targeted pollutant, and groundwater transport. Wastewater plumes are difficult to locate and intercept with an intervention. Treatment is less efficient where pollution is dilute, dispersed or not recovered. Meeting water quality goals in the near to moderate term may require deploying a suite of approaches to simultaneously deal with legacy, recurring and new sources. Watershed-scale planning and commensurate implementation of centralized, decentralized, engineered and nature-based solutions for wastewater pollution will be needed to reduce total loading and repair water resources.

## 5. Conclusions

Enhanced onsite wastewater treatment can support water quality goals in areas with impairments or vulnerability. Enhanced Innovative/Alternative (I/A) septic systems that include a woodchip bioreactor can remove more than 90% of nitrogen (N) loads from effluent and mitigate other pollutants. Partnerships are needed to field test technologies, build capacity to manage them and ensure performance, and increase user access and experience. In this demonstration study, partners worked to strategically deploy and monitor two promising options at neighboring residences in Barnstable, Massachusetts (Cape Cod). Monitoring data indicated high performance, defined as effluent TN < 10 mg/L, a new goal for best available N-reducing technologies in the state. Reliability was also high when use and operating conditions were consistent with technologic design. Enhanced I/A septic systems on individual parcels can significantly reduce wastewater N loads where clustered or centralized treatment is not available.

Excessive N loading and its cascading consequences are well quantified and understood on Cape Cod, where overreliance on conventional septic systems has degraded water quality, aquatic habitat, economic and culturally significant activities. Water resource impairments have motivated extensive planning and a range of intensive mitigation efforts. In other parts of the country with existing or expanding unsewered development, the slow degradation of water resources may not be as well recognized or documented and, without investigation, may be obscure to the public. Enhanced septic systems and other proactive measures to control loading can contribute to preventing damage. New or unfamiliar approaches to nutrient management involve trade-offs in terms of cost, complexity, time, social acceptability, and environmental impact, as does the status quo. Public awareness, engagement, and commitment to protecting water quality are needed to make durable progress on reducing total pollutant loads.

## Supplementary Material

Supplement1

## Figures and Tables

**Table 1:** Laboratory sample handling, analysis, and reporting limits for water quality indicators measured in the wastewater of 14 Innovative/Alternative septic systems in Barnstable (Cape Cod), Massachusetts, a cohort comprised of two models that incorporate woodchip bioreactors to enhance N removal.

Parameter	Method^a,b^	Approach	RL^c^	Unit	Sample handling
Alkalinity	SM 2320-B	titration	2	mg/L as CaCO_3_	250-mL plastic, ice
Ammonia (NH_3_)	EPA 350.1	colorimetry	0.25	mg/L as N	250-mL plastic, ice, H_2_SO_4_
Biochemical oxygen demand (BOD)	SM 5210 B	5-day incubation	2	mg/L	1000-mL plastic, ice
Nitrate (NO_3_^−^)	EPA 300.0	ion chromatography	0.1	mg/L as N	250-mL plastic, ice
Nitrite (NO_2_^−^)	EPA 300.0	ion chromatography	0.05	mg/L as N	250-mL plastic, ice
Total Kjeldahl Nitrogen (TKN)	EPA 351.2	colorimetry	0.25	mg/L as N	250-mL plastic, ice, H_2_SO_4_
Total phosphorus (TP)	EPA 365.1	colorimetry	0.01	mg/L	250-mL plastic, ice
Total suspended solids (TSS)	SM 2540 D	gravimetry	4	mg/L	250-mL plastic, ice

a Most methods are available for download from the National Environmental Methods Index (www.nemi.gov).

b SM = Standard Methods, see http://standardmethods.org/.

c RL = Reporting Limit.

**Table 2:** Summary values for nutrient concentrations by site and port, and wastewater flow rates for 13 Innovative/Alternative septic systems in Barnstable (Cape Cod), Massachusetts, a cohort comprised of two models that incorporate woodchip bioreactors to enhance N removal. Sites are described in section 2.1.

		Concentration (mg/L)															Flow (gpd)				
		Influent					Effluent					Lysimeter									
Site	Param.	Min	Med.	Mean	Max	n	Min	Med.	Mean	Max	n	Min	Med.	Mean	Max	n	Min	Med.	Mean	Max	n
A	TN	51	84.7	89.9	220.9	24	1.4	4.4	6.8	33.4	24	0.5	2.5	6	31.9	24	0.1	129.3	128.5	298.3	594
	TP	4.8	7.4	8.2	21	24	1.8	5.7	5.8	9.8	24	–	–	–	–	0					
B	TN	66.8	88	87.8	114.2	21	0.2	1.7	4.1	47.4	21	0.8	1.7	2.8	14.1	13	0	75.5	92.1	1040.8	594
	TP	5.8	9	8.9	12	21	1.3	5.1	5.3	8.4	21	–	–	–	–	0					
C	TN	51.4	87.8	90.2	131.6	15	0.4	1.4	6.4	44.1	25	–	–	–	–	0	0	82.6	263.7	1909.6	592
	TP	6.4	9.4	9.4	12	15	2.8	5.2	5.3	7.5	25	–	–	–	–	0					
D	TN	75.4	93.7	92.9	131	25	1.9	3.7	4	9.3	25	–	–	–	–	0	33.9	145.6	160.2	647.7	592
	TP	4.2	11	11.3	16	25	6.7	10	10.1	14	25	–	–	–	–	0					
E	TN	82.9	130.5	125.9	171.2	14	0.4	7.2	19.9	100.8	24	–	–	–	–	0	–	–	–	–	0
	TP	9.3	13.5	13.8	23	14	7.3	11	10.9	16	24	–	–	–	–	0					
F	TN	72.8	148.8	143.8	181	15	1.5	4.9	23.1	121.4	23	–	–	–	–	0	0	69.3	78.3	373.8	592
	TP	6.4	14	13.6	18	15	8.3	12	12.1	16	23	–	–	–	–	0					
G	TN	53.9	78	77.1	100.5	16	0.4	2.4	2.5	4.5	17	–	–	–	–	0	0	59.8	73.5	2098.2	532
	TP	2.6	8.2	7.6	10	16	3.4	4.6	5.4	9.6	17	–	–	–	–	0					
H	TN	72.2	84.7	90.9	121.9	15	1.4	2.9	4.9	18.7	17	–	–	–	–	0	0	149	191.8	2025.3	564
	TP	7.5	9.3	10	13	15	4.5	6.3	6.5	9.4	17	–	–	–	–	0					
I	TN	61.8	90.2	113.7	420.8	15	1.8	2.8	4.6	16.8	17	2.2	4.4	7.1	23.9	17	49.2	143.2	182.7	1394	565
	TP	6.9	10	9.8	14	15	4	5.9	5.7	7.6	17	–	–	–	–	0					
J	TN	70.3	169.1	163	241	15	1.2	2.8	6.2	34	17	–	–	–	–	0	0	57.7	67.3	1216.4	565
	TP	11	18	18.7	26	15	6.4	8	8	11	17	–	–	–	–	0					
K	TN	75.9	99	107.7	161.6	15	1.4	2.9	7.2	72.8	17	1.4	5.1	12.7	101	17	0	25.5	60.6	828	565
	TP	9.5	14	15.1	34	15	4.5	10	8.9	14	17					0					
L	TN	29.8	39.6	40.8	57.1	15	0.5	1.2	1.4	4.1	17	0.5	1.4	2	6.1	16	0.1	80.3	97	620.8	585
	TP	3.3	4.9	5	7.5	15	2	3.3	3.3	4.4	17					0					
M	TN	41.5	46.3	47.4	56.4	14	30.1	36	36.6	43.8	11	–	–	–	–	0	202	368.8	390.4	851.9	497
	TP	4.3	5.4	5.8	9.2	12	1.1	2.4	2.2	3.1	11	–	–	–	–	0					

TN = total nitrogen, TP = total phosphorus, mg/L = milligrams per liter, gpd = gallons per day.

**Table 3:** Summary of sample values, by port and parameter, in the wastewater of 13 Innovative/Alternative septic systems in Barnstable (Cape Cod), Massachusetts, a cohort comprised of two models that incorporate woodchip bioreactors to enhance N removal.

Parameter	Influent					Effluent					Lysimeter				
	min	median	mean	max	n	min	median	mean	max	n	min	median	mean	max	n
alkalinity (mg/L as CaCO_3_)	140	340	348.4	660	75	1	110	115	520	92					0
BOD_5_ (mg/L)	21	170	174.8	600	77	1	1	12.1	130	93					0
DO (mg/L)	0.1	0.4	0.6	12.5	217	0	8.9	8.5	12.9	254	0.1	8	7.2	12.2	88
NH_3_ (mg/L as N)	20	75	79.4	170	77	0.1	0.1	5.3	82	92					0
NO_2_^−^ (mg/L as N)	0	0	0.2	26	219	0	0	0.1	2.8	255	0	0	0.1	1.9	87
NO_3_^−^ (mg/L as N)	0	1	1.4	16	219	0	0.8	3.3	46	255	0	1	2.5	31	87
pH [−]	5.7	6.8	6.8	7.8	218	3.9	7.4	7.2	8.4	254	4.1	7	7	8.2	88
SpC (uS/cm)	237	859.9	885.3	2358.2	218	1.2	491.1	523	1277.2	254	0.2	431.9	440.9	1212.6	87
temperature (°C)	5.8	17.2	17	26.1	218	5.3	16	15.4	24.9	254	5.7	15.6	14.5	23.2	88
TKN (mg/L as N)	28	88	95.4	420	219	0.1	1.6	5.9	120	255	0.1	1.2	3.6	100	88
TN (mg/L)	29.8	89.1	97	420.8	219	0.2	3	9.3	121.4	255	0.5	2.7	6.3	101.1	87
TP (mg/L)	2.6	9.5	10.5	34	217	1.1	6.6	7.2	16	255					0
TSS (mg/L)	6.7	64	78.8	250	75	1	7.8	23.8	180	92					0
turbidity (NTU)	5	61.6	168.6	2873.8	217	0	4	13.5	461.1	238	0	3.4	34.8	1137.1	72

BOD_5_ = 5-day biochemical oxygen demand, DO = dissolved oxygen, TSS = total suspended solids, NH_3_ = ammonia, NO^−^_2_ = nitrite, NO_3_^−^ = nitrate, TKN = total Kjeldahl, SpC = specific conductance, TN = total nitrogen, TP = total phosphorus, NTU = nephelometric turbidity units.

## Data Availability

Dataset is available at: https://doi.org/10.23719/1529539.

## Appendix A. Supplementary data

Supplementary data to this article can be found online at https://doi.org/10.1016/j.jenvman.2024.122737.
