# Supplementary material for: Reducing wastewater nitrogen loading by >90% with carbon-amended septic systems: A field demonstration in Barnstable (Cape Cod), Massachusetts
Source: J Environ Manage. Author manuscript; Available in PMC 2025 Nov 1. (PMC11568896; doi:10.1016/j.jenvman.2024.122737)
Supplement: Supplement1 [file NIHMS2029942-supplement-Supplement1.docx]

­

Supplementary materials for:

“Reducing wastewater nitrogen loading by >90% with carbon-amended septic systems: a field demonstration in Barnstable (Cape Cod), Massachusetts”

Laura E. Erban^1*^, Sara K. Wigginton^2^, Brian Baumgaertel^2^, Bryan Horsley^2^, Timothy D. McCobb^3^, Zenas Crocker^4^, Scott Horsley^5^, Timothy R. Gleason^1^

^1^ U.S. Environmental Protection Agency, Office of Research and Development, Atlantic Coastal Environmental Sciences Division, Narragansett, RI

^2^ Massachusetts Alternative Septic System Test Center, Sandwich, MA

^3^ U.S. Geological Survey. New England Water Science Center, Northborough, MA

^4^ Barnstable Clean Water Coalition, Osterville, MA

^5^ Horsley Consulting, Cotuit, MA

* corresponding author

*Disclaimer*: The views expressed in this supplementary material are those of the authors and do not necessarily represent the views or policies of the U.S. Environmental Protection Agency. Any mention of trade names, products, or services does not imply an endorsement by the U.S. Government or the U.S. Environmental Protection Agency. The U.S. Environmental Protection Agency does not endorse any commercial products, services, or enterprises.

1. *Difference in outcomes among sampling ports and periods*

The following figures illustrate additional results from monitoring wastewater in 13 Innovative/Alternative septic systems in Barnstable (Cape Cod), Massachusetts, a cohort comprised of two models that incorporate woodchip bioreactors to enhance N removal. The figures are provided in the order referenced in the main manuscript. The first is a comparison of the smaller set (n = 5) of paired effluent and lysimeter samples. Over the full monitoring record, mean total nitrogen (TN) were higher in lysimeter than in effluent samples at 4 of 5 sites (Fig. S1). Differences were not statistically significant (per the Wilcoxon signed rank test). At these low concentrations, noise in the data become more influential. Effluent quality is variable in time, there is a lag in time between effluent and lysimeter samples, and the latter may also be influenced by precipitation or evaporation in the leach field and pan. There is clearly no evidence in these data of additional N removal after the septic system enhancement, which is consistent with expectations given the low concentrations in effluent and composition of these leach fields, made of empty concrete chambers.


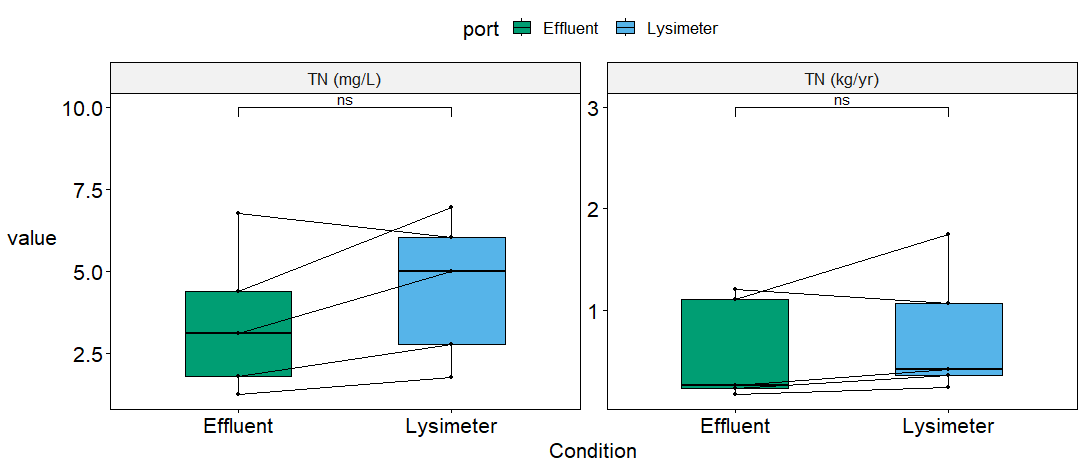


**Figure S1**. Concentrations (*left*) and estimated loads (*right*) of TN in paired effluent and lysimeter wastewater samples from 13 Innovative/Alternative septic systems in Barnstable (Cape Cod), Massachusetts, a cohort comprised of two models that incorporate woodchip bioreactors to enhance N removal. Each point is a mean value for the full monitoring record and sampling port, with lines connecting values paired by system. Note the differences in y-scale between plots. The statistical significance of differences across the group is indicated above the brackets (ns = not significant).

The complete dataset covers a variable timespan for samples per system, depending on installation date. Restricting the monitoring record to the most recent full year of collection (2023), when samples were collected for all systems, gives similar results as for the complete dataset. The plot below shows no visually meaningful differences in outcomes for any particular system or the group as a whole, and no difference in the overall levels of significance.


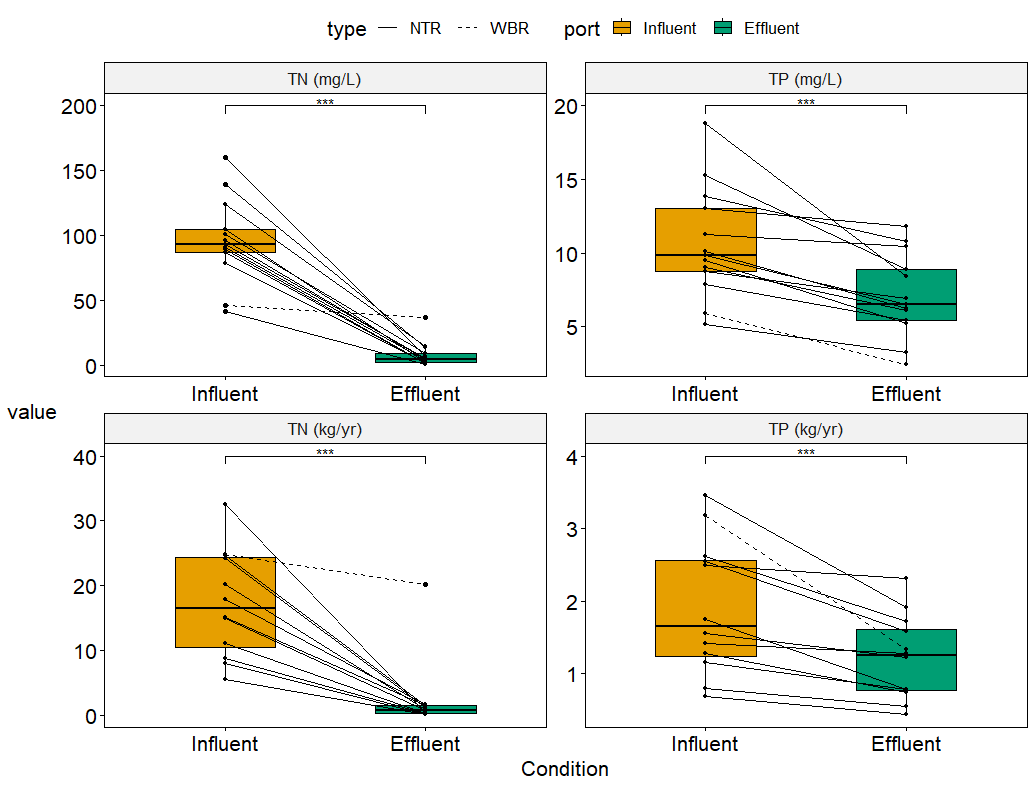


**Figure S2**. Nutrient concentrations (*top*) and estimated loads (*bottom*) of total nitrogen TN and total phosphorus TP before and after enhanced treatment for 12 Innovative/Alternative septic systems in Barnstable (Cape Cod), Massachusetts. The cohort is comprised of two models that incorporate woodchip bioreactors to enhance N removal. Only sites with flow meters and data, needed for load estimates, are presented for consistency within the figure. Sample data are restricted to the year 2023, for strict temporal overlap and balanced sample size. Each point is a mean value for the year and sampling port, with lines connecting values paired by system. Note the differences in y-scale between TN and TP plots. The dashed lines correspond to the nonproprietary system. The statistical significance of differences between influent and effluent values across the group is indicated above the brackets (***, p < 0.001).

1. *Exploratory data analysis (EDA)*

Throughout the monitoring period, data were evaluated by sampling event, system and parameter. Given the number of systems (13), water quality parameters (14), sampling ports (3), continuous flow measurements and loading estimates made from them, there are many ways to view, evaluate and summarize the dataset. Figures provided here show some of the key patterns discussed in the main manuscript.


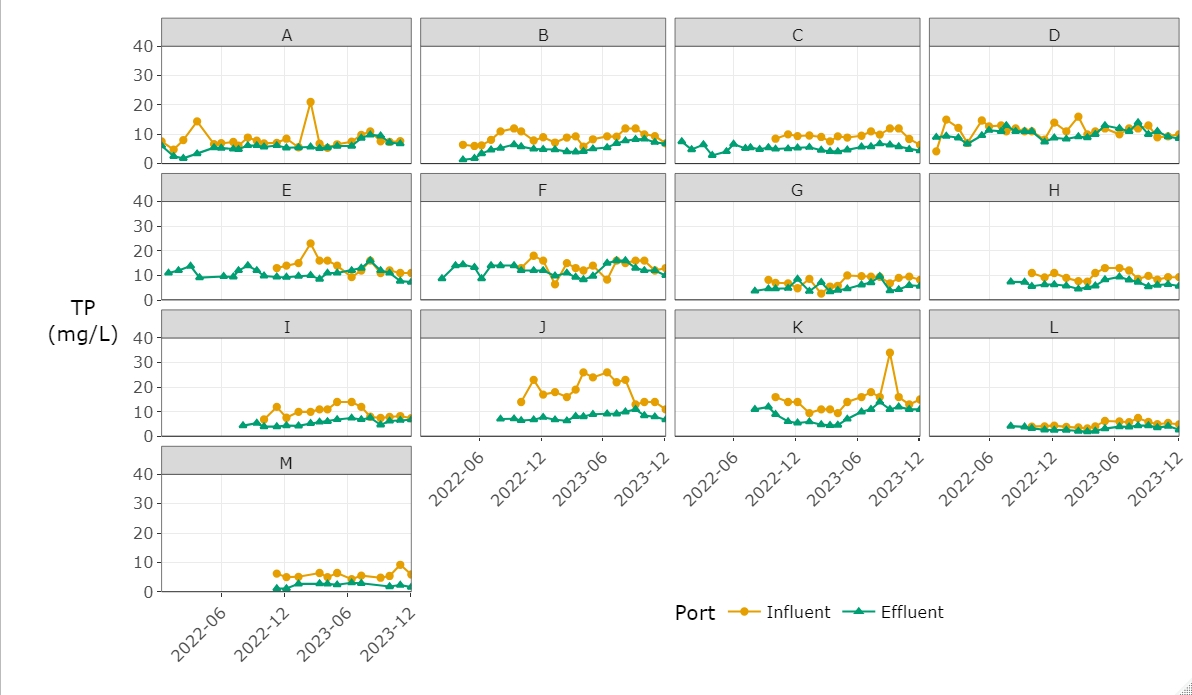


**Figure S3**. Total phosphorus (TP) concentrations over time, by site and sampling port, in wastewater from 13 Innovative/Alternative septic systems in Barnstable (Cape Cod), Massachusetts, a cohort comprised of two models that incorporate woodchip bioreactors to enhance N removal. Lysimeter samples were not analyzed for phosphorus.


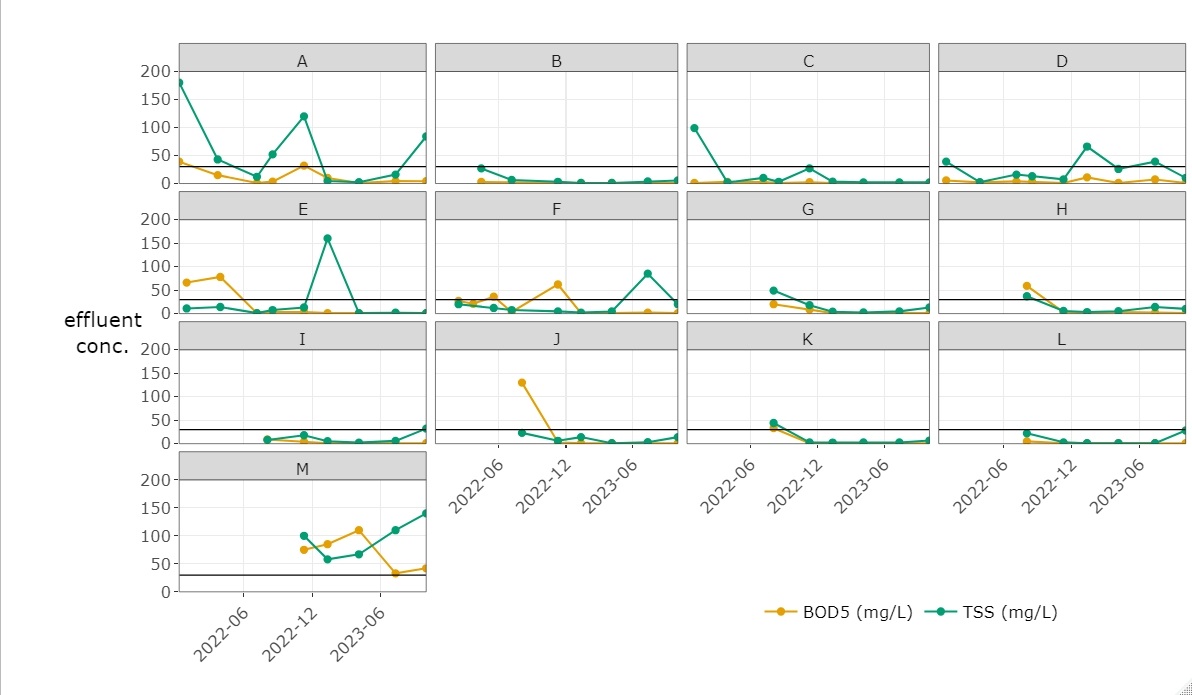


**Figure S4**. 5-day biochemical oxygen demand (BOD_5_) and total suspended solids (TSS) concentrations over time, by site and sampling port, in wastewater from 13 Innovative/Alternative septic systems in Barnstable (Cape Cod), Massachusetts, a cohort comprised of two models that incorporate woodchip bioreactors to enhance N removal. Lysimeter samples were not analyzed for these parameters. The regulatory effluent limit of 30 mg/L is in indicated with a black line in plots.


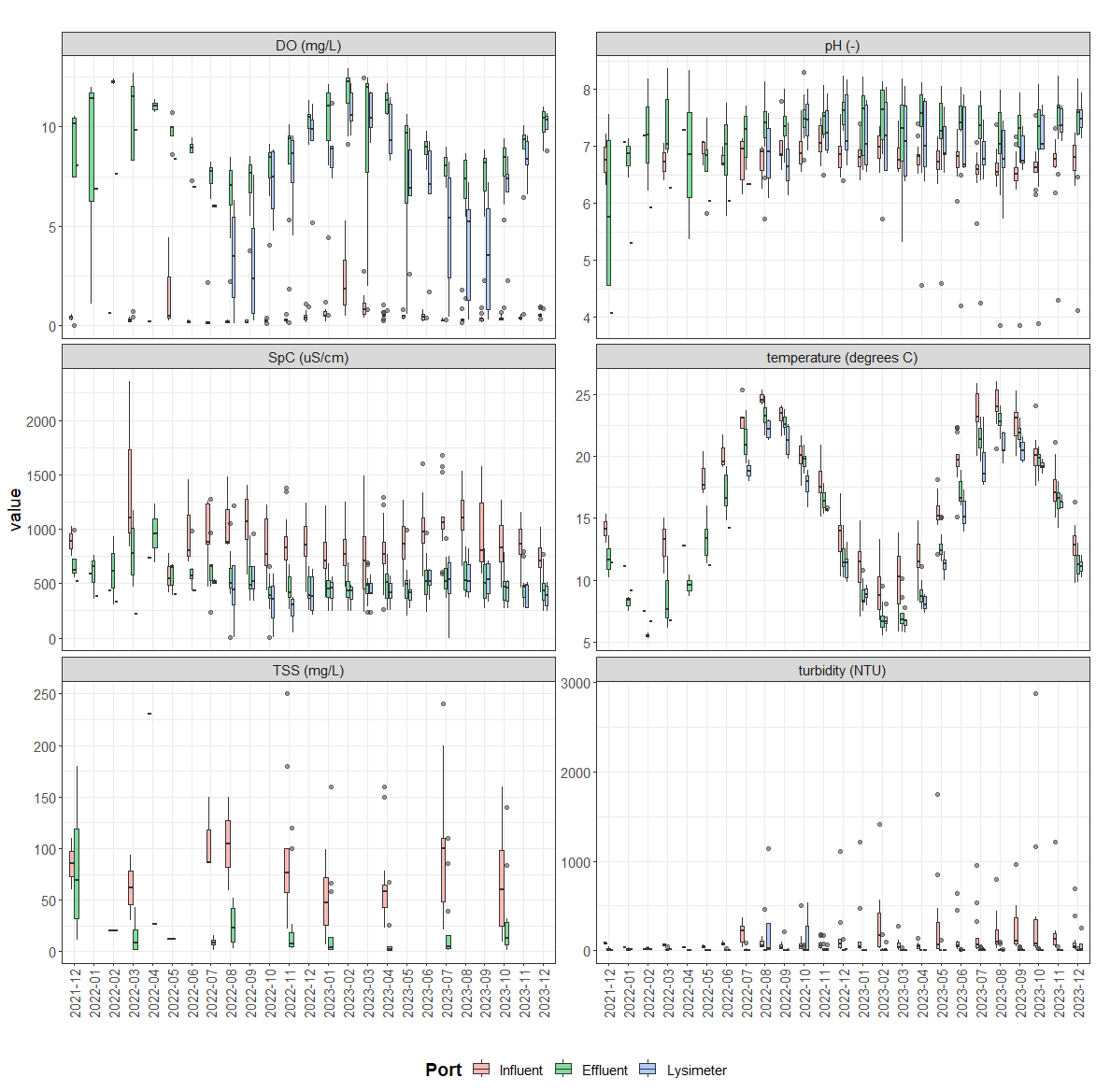


**Figure S5**. Field parameters and total suspended solids levels (TSS) over time across sites, by sampling port, in wastewater from 13 Innovative/Alternative septic systems in Barnstable (Cape Cod), Massachusetts, a cohort comprised of two models that incorporate woodchip bioreactors to enhance N removal. Box plots depict the minimum, first quartile, median, third quartile, and maximum, with outliers depicted as single points. DO = dissolved oxygen, TSS = total suspended solids, SpC = specific conductance, NTU = nephelometric turbidity units.


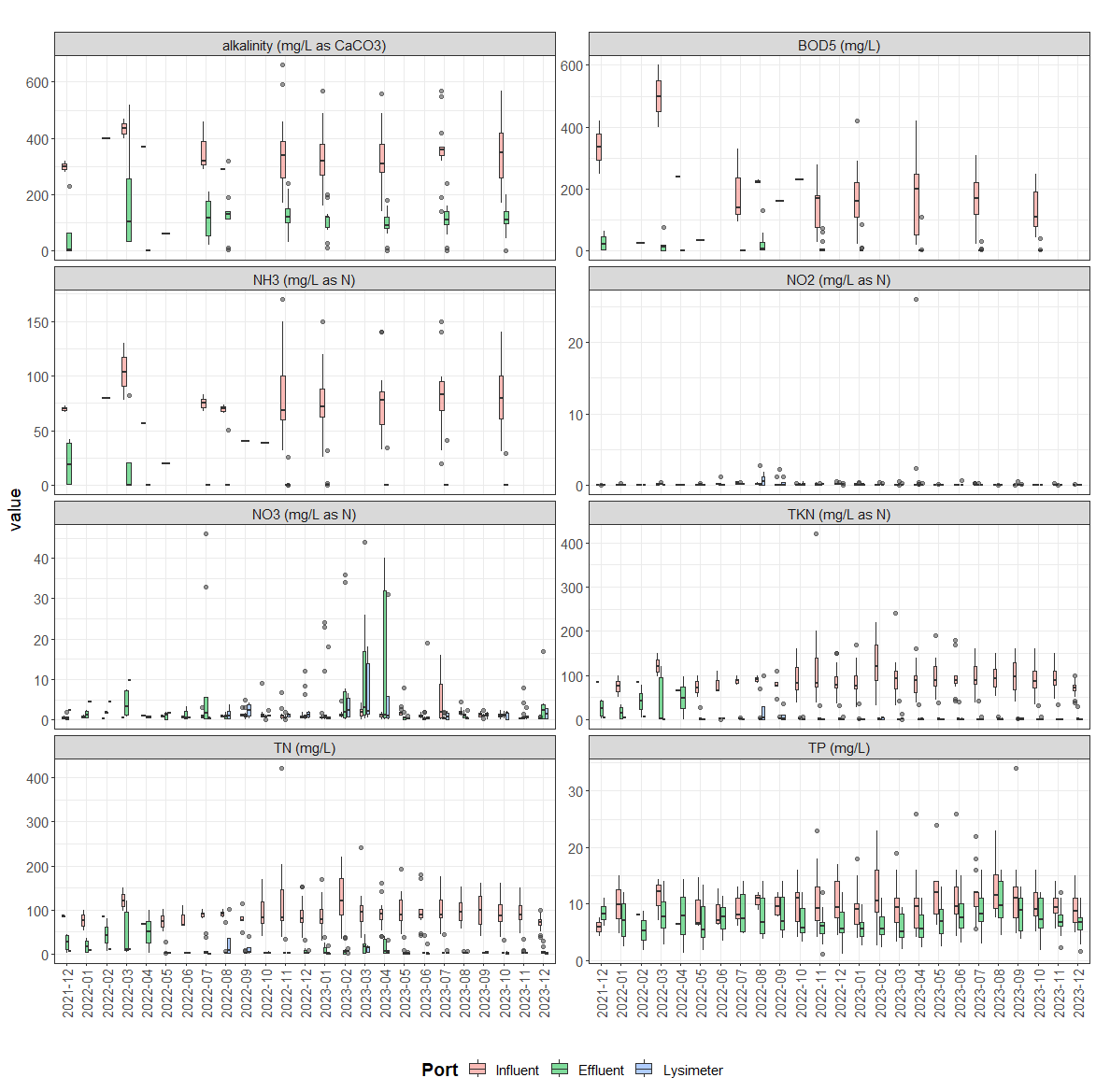


**Figure S6**. Additional laboratory parameters over time across sites, by sampling port, in wastewater from 13 Innovative/Alternative septic systems in Barnstable (Cape Cod), Massachusetts, a cohort comprised of two models that incorporate woodchip bioreactors to enhance N removal. Box plots depict the minimum, first quartile, median, third quartile, and maximum, with outliers depicted as single points. BOD_5_ = 5-day biochemical oxygen demand, NH_3_ = ammonia, NO_2_^-^ = nitrite, NO_3_^-^ = nitrate, TKN = total Kjeldahl, TN = total nitrogen, TP = total phosphorus.


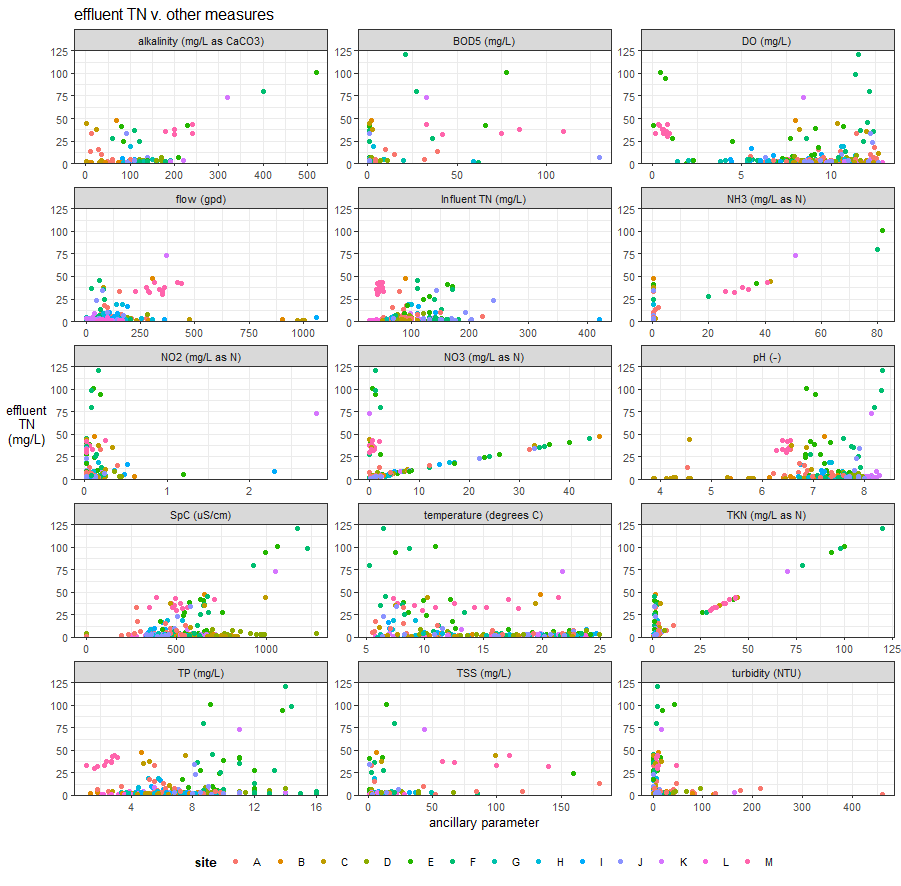


**Figure S7**. Effluent total nitrogen (TN) concentrations (mg/L) versus ancillary flow and water quality measures in wastewater from 13 Innovative/Alternative septic systems in Barnstable (Cape Cod), Massachusetts, a cohort comprised of two models that incorporate woodchip bioreactors to enhance N removal. Note that the x-axis ranges vary by parameter. BOD_5_ = 5-day biochemical oxygen demand, DO = dissolved oxygen, gpd = gallons per day, TSS = total suspended solids, NH_3_ = ammonia, NO_2_^-^ = nitrite, NO_3_^-^ = nitrate, TKN = total Kjeldahl, SpC = specific conductance, TP = total phosphorus, NTU = nephelometric turbidity units.
